# Environmental Issues and Neurological Manifestations Associated with COVID-19 Pandemic: New Aspects of the Disease?

**DOI:** 10.3390/ijerph17218049

**Published:** 2020-11-01

**Authors:** Luigi Bellocchio, Ioana Roxana Bordea, Andrea Ballini, Felice Lorusso, Denisa Hazballa, Ciro Gargiulo Isacco, Giuseppina Malcangi, Alessio Danilo Inchingolo, Gianna Dipalma, Francesco Inchingolo, Prisco Piscitelli, Giancarlo Logroscino, Alessandro Miani

**Affiliations:** 1 INSERM, U1215 NeuroCentre Magendie, Endocannabinoids and Neuroadaptation, University of Bordeaux, 33000 Bordeaux, France; gigett@gmail.com; 2 Department of Oral Rehabilitation, Faculty of Dentistry, Iuliu Hațieganu University of Medicine and Pharmacy, 400012 Cluj-Napoca, Romania; 3 Department of Biosciences, Biotechnologies and Biopharmaceutics, Campus Universitario “Ernesto Quagliariello” University of Bari “Aldo Moro”, 70125 Bari, Italy; andrea.ballini@uniba.it; 4 Department of Precision Medicine, University of Campania “Luigi Vanvitelli”, 80138 Naples, Italy; 5 Department of Medical, Oral and Biotechnological Sciences, University of Chieti-Pescara, 66100 Chieti, Italy; drlorussofelice@gmail.com; 6 Kongresi Elbasanit, Rruga: Aqif Pasha, 3001 Elbasan, Albania; denisahazballa@gmail.com; 7 Embryology and Regenerative Medicine and Immunology at Pham Chau Trinh University of Medicine Hoi An, Ho Chi Minh 70000, Vietnam; drciroisacco@gmail.com; 8 Regenerative Medicine and Metabolic Disorders at Department of Interdisciplinary Medicine, University of Medicine Aldo Moro, 70121 Bari, Italy; 9 Department of Interdisciplinary Medicine, University of Medicine Aldo Moro, 70121 Bari, Italy; giuseppinamalcangi@libero.it (G.M.); ad.inchingolo@libero.it (A.D.I.); giannadipalma@tiscali.it (G.D.); francesco.inchingolo@uniba.it (F.I.); 10 Staff UNESCO Chair on Health Education and Sustainable Development, Federico II University, 80138 Naples, Italy; 11 Department of Basic Medical Sciences, Neuroscience and Sense Organs, University of Bari Aldo Moro, 70121 Bari, Italy; giancarlo.logroscino@uniba.it; 12 Department of Environmental Sciences and Policy, University of Milan, 20068 Milan, Italy; alessandro.miani@unimi.it; 13 Italian Society of Environmental Medicine, SIMA, 20068 Milan, Italy

**Keywords:** neurological symptoms, environment, COVID-19, stroke, encephalitis, delirium

## Abstract

Coronavirus (SARS-CoV-2) emerged in China in December 2019 and rapidly caused a global health pandemic. Current evidence seems to suggest a possible link with ecosystem disequilibrium and even air pollution. The primary manifestations affect respiratory and circulatory systems, but neurological features are also being reported through case reports and case series. We summarize neurological symptoms and complications associated with COVID-19. We have searched for original articles published in PubMed/Medline, PubMed Central and Google Scholar using the following keywords: “COVID-19”, “Coronavirus”, “pandemic”, “SARS-COV-2”, “neurology”, “neurological”, “complications” and “manifestations”. We found around 1000 publications addressing the issue of neurological conditions associated with COVID-19 infection. Amongst those, headache and dizziness are the most common reported symptoms followed by encephalopathy and delirium, while the most frequent complications are cerebrovascular accidents, Guillain–Barré syndrome, acute transverse myelitis, and acute encephalitis. Specific symptoms affecting the peripheral nervous system such as hyposmia and dysgeusia are the most common manifestations recorded in the selected studies. Interestingly, it was noted that these kinds of neurological symptoms might precede the typical features, such as fever and cough, in COVID patients. Neurological symptoms and complications associated with COVID-19 should be considered as a part of the clinical features of this novel global pandemic.

## 1. Introduction

The novel Coronavirus (COVID-19) pandemic started in Wuhan city, located in the Chinese province of Hubei, at the end of 2019 [1,2]. An undetermined etiology started to underlie unusual cases of pneumonia as reported by Chinese authorities [3]. The Wuhan market was initially identified as a unique cluster for all cases. In March 2020, COVID-19 became the new pandemic according to World Health Organization (WHO) [4]. As of the 15 September 2020, there are almost 30 million cases of COVID-19 with more than 900,000 deaths worldwide [5]. 

Studying COVID-19 has greatly attracted basic scientists, researchers and medical doctors worldwide, as represented by the amount of research and articles on COVID-19, which is something that has never been seen before. Since the beginning of 2020, almost thousands of manuscripts have been submitted, and the majority of them have been published, as reported by recent estimations [6,7,8]. So far, all aspects of the disease including manifestations, pathology and transmission, prevention and management strategies have been covered by huge sets of theoretical and experimental data [9,10,11]. It is known that particulate matter (PM) fractions (e.g., PM2.5 and PM10) serve as carriers for several chemical and biologic pollutants, including viruses, which may be absorbed, through coagulation processes, on particulate matter (composed by solid and/or liquid particles) [12]. PM becomes a substrate allowing long term persistence of viruses in the atmosphere for hours or days and adsorbed biologic pollutants may be subjected to diffusion into the atmosphere and transported for long distances [13]. Viral inactivation depends on selected environmental parameters, on one hand both high temperature and solar radiation are able to speed up the inactivation rate, and on the other hand high relative humidity may promote the diffusion rate. Recently published scientific studies have already highlighted the relationship between viral diffusion among the exposed population and particulate matter concentration into the atmosphere [13,14,15,16,17]. Finally, SARS-CoV-2 RNA has been found on particulate matter in Bergamo, the epicenter of the COVID-19 outbreak in Italy [18]. 

There is also increasing evidence that air pollution could influence the COVID-19 outbreak progression, enhancing the host susceptibility to viral infection by independently increasing the baseline risk of cardiovascular events and complications, chronic obstructive pulmonary diseases (COPD), and other conditions that are known to increase the severity of the infections especially in elderly people [19,20,21,22]. 

The respiratory and cardiovascular systems have been reported as the main targets affected in COVID-19. However, in several cases, neurological symptoms (ranging from hypogeusia/hyposmia to headache, dizziness and neuralgia) and severe neurological complications (including encephalopathy, impaired consciousness, skeletal muscular injury and acute cerebrovascular diseases) often follow COVID-19 infection [23,24,25]. In this editorial, we have reviewed the complications and neurological symptoms that have been reported during the COVID-19 pandemic. This will help update the physicians, especially neurologists working with suspected COVID-19 cases, in which neurological symptoms and complications are part of a global health threat.

## 2. Materials and Methods 

We have searched for original articles published in Google Scholar, PubMed/Medline and Central. Our research used the following keywords: “Coronavirus and COVID-19”, “SARS-COV-2”, “neurology”, “complications” and “manifestations”. We found around 780 results with PubMed and 226 results with Google Scholar. The type of publications were mostly clinical trials and case reports, but we also found some reviews and systematic analyses. After screening for risk of bias assessment [26] according to the PRISMA statement (Preferred Reporting Items for Systematic Reviews and Meta-Analyses) [27], around 16 papers were included and used to describe the alteration of the nervous system by COVID-19 reported in this review. 

## 3. Results

### 3.1. Mechanisms of Cns Invasion and Damage

Although a complete experimental characterization is available for COVID-19, this disease is considered to be generated from a mutated form of two previously known pathologies caused by coronaviruses, such as Middle East Respiratory Syndrome (MERS) and the Severe Acute Respiratory Syndrome (SARS) viruses [28,29]. Therefore, SARS-CoV-2 shares similar biological features and behaviors compared to the aforementioned microorganisms [28,29,30]. Coronavirus primarily infects respiratory epithelial cells and indeed they do not show neuronal tropism [28]. These biological agents use the angiotensin converting enzyme-2 receptor (ACE 2) for attachment to cells and subsequent internalization. After cellular penetration, the viral RNA is released in the cytoplasm, translated, and replicated. The viruses, derived from the formation of envelope proteins and the incorporation of RNA into them, are then released into circulation [31].

ACE 2 receptors are not only expressed by epithelial cells, as they also have been found in glial cells from brain and spinal neurons [29,30,31]. Thus, it is not surprising that neuronal tissue might also be a target of the cytotoxic properties of SARS-CoV-2. Indeed, evidence from pre-clinical studies in mice have shown that coronavirus possesses a particular transfer mechanism that allows the retrograde entry to the brain, starting from the cribriform bone and/or the olfactory epithelium. Thus, viral particles are able to reach the brain in seven days’ time, at least in rodents [31]. On the other hand, disruption of the blood brain barrier underlies the viremic phase of the disease; in this scenario, a further viral spillover, directly to the brain, has been postulated. Moreover, to gain entry to the central nervous system (CNS), the invasion of peripheral nerve terminals (especially traveling through the synapse connected rout) by SARS-CoV-2 might represent another putative weapon for the virus [31]. Given the similarities between COVID-19 and Severe Acute Respiratory Syndrome (SARS CoV), it is not unlikely that the new coronavirus might follow the same pathways discussed above to penetrate the brain. For readers interested in host and virus interaction, the following reviews might be of interest for readers interested in knowing more [30,31,32]. 

COVID-19 has been shown to cause neurological damage by two likely mechanisms: (a) *hypoxic brain injury:* systemic hypoxia can be caused by severe pneumonia, which leads to the damage of several organs including the brain. Hypercarbia, hypoxia and anaerobic metabolism, associated with peripheral vasodilatation, are main players in this field, which eventually lead to the accumulation of toxic compounds. Thus, subsequent neuronal swelling and brain edema will ultimately result in neurological injury [10,33]; (b) *Immune mediated injury:* in this case, the main factor responsible is the cytokine storm, characterized by increased levels of inflammatory mediators and activation of macrophages, T lymphocytes and endothelial cells. This kind of response highlights the majority of viral infections. Indeed, interleukin 6 has been known to cause vascular leakage, coagulation cascade and complement activation. The cytokine storm and vascular leakage finally leads to disseminated intravascular coagulation and will finish to damage the interested organs (e.g., lungs and heart primarily, CNS afterwards) [34,35].

### 3.2. Neurological Manifestations of COVID-19

Two case series were published in which authors provide a detailed listing of neurological symptoms and complications that accompanied the progression of COVID-19 in patients. First, a retrospective case series published from China [23]. Patients were divided in two groups, the severely ill group and the non-severely ill group were represented, respectively, by 41.1% and 58.9% of the subjects. Patients belonging to the severely ill group showed more co-morbid conditions (such as hypertension) and were significantly older. Surprisingly, the severely ill group was characterized by less typical symptoms of coronavirus such as fever. However, severe cases, when compared with non-severe cases, reported significantly more nervous system symptoms, with dizziness and headache amongst the most commonly described [23]. The second prospective case series was conducted on 58 patients from France [24]. The subjects were an average of 63 years old and presented a higher percentage of CNS complications when compared to the study mentioned before. Among the most common symptoms were agitation and confusion, which were identified by the use of the confusion assessment method for intensive care unit (CAM-ICU) scale. At the time of discharge, signs of cortico-spinal damage were present and, interestingly, were accompanied by dysexecutive syndrome [24]. As will be summarized and discussed below, it is worth keeping in mind that the spectra of neurological manifestations and complications of COVID-19 is not only limited to the brain but also interests peripheral nerves.

### 3.3. Central Nervous System Manifestations

#### 3.3.1. Encephalopathy

Although little is known about the details and the diagnostic criteria, the first report of headache and encephalopathy associated with COVID-19 was reported in a consistent percentage of patients from the Chinese retrospective case series described above [23]. Neurological symptoms were also described in two case reports from the USA(United States of America) . Indeed, a 72 and 74 year-old male, respectively, with a past medical history of several age-related diseases presented with a fever and cough [36,37]. Initial diagnostic procedures excluded serious issues and one of the patients was even sent home. Patients went back to the hospital due to a worsening of symptoms. Chest X-rays and brain computer tomograph (CT) scans were suggestive of pneumonia and previous stroke, respectively, [36,37] and the patients also presented with a headache and altered mental status. Although no infection was detected in the crebrospinal fluid (CSF) of both patients, they tested positive for COVID-19 and developed respiratory failure. Two other Chinese retrospective studies highlighted hypoxic encephalopathy amongst clinical features, with an incidence significantly lower after recovery [38,39].

#### 3.3.2. Acute Hemorrhagic Necrotizing Encephalopathy (ANE) 

The first case of COVID-19-associated acute hemorrhagic necrotizing encephalopathy (ANE) was reported in the USA [35,36]. A middle-aged female patient with a three-day history of cough, fever, and altered mental status was reported by Poyiadji and colleagues. Although negative for other reported pathogens, the patient tested positive for COVID-19. Authors also reported symmetric hypo-attenuation within the bilateral medial thalami and brain magnetic resonance imaging (MRI) demonstrated hemorrhagic rim [40,41]. ANE is a rare complication associated with several viral infections of the respiratory tract. One of the proposed physio-pathological mechanisms relies on the cytokine storm with subsequent brain damage and impairment of the integrity of the blood brain barrier.

#### 3.3.3. Cerebrovascular Accidents (CVA) 

The first evidence of COVID-19-associated CVA came from an Iranian case report from Sharifi and colleagues. Authors reported a case of intracranial bleeding resulting in CVA associated with successful COVID-19 detection [36,37,42,43]. The patient was semi-conscious and had a history of fever and cough when admitted to the emergency room (Glasgow Coma Scale 7/15) with bilateral extensor planter response and signs of viral pneumonia detected by a chest CT scan. A brain CT scan revealed intraventricular and subarachnoid extension accompanied with a massive hemispheral bleed with interestingly no signs of previous pathological conditions that could have caused this event [42,43]. A possible dysregulation of the cerebral blood flow as well as the ACE 2 receptors and the sympathoadrenal system could have resulted in the bleed observed in the brain despite the near normal blood pressure [42,43]—another aspect very difficult to explain according to the authors.

Two case series specifically reporting brain symptoms of COVID-19 further provide evidence of CVA associated with the disease. Six cases of CVA were reported in their cohort of 214 patients from Mao and colleagues [17,23]. There were five ischemic cases of stroke and one hemorrhagic. On the other hand, three cases of ischemic strokes (detected by neuroimaging looking for encephalopathy without other focal neurological signs) were also reported in the French cohort [24], indicating that symptoms were probably not well characterized due to the encephalopathy. However, this case provides a key example of how neuroimaging can be useful in the complete clinical evaluation of COVID-19 patients, pointing out the need for more evidence to fully highlight a causal relationship between COVID-19 and strokes. Stroke generally occurs after 2–3 weeks in the natural history of COVID-19 infection. Sometimes, however, stroke onset is in the early phase. A series of five patients with large vessel disease as initial symptoms of COVID-19 has been described in New York City [44]. 

#### 3.3.4. Encephalitis 

Seizures and unconsciousness, associated with fever and other symptoms, were reported in a Japanese young man, which has become the first confirmed case of viral encephalitis associated with COVID-19 [38,45]. The patient presented with a normal brain CT scan and with neck stiffness and patchy pneumonia. COVID-19 was detected in the CSF sample but not in nasopharynx secretions. Hyperintensity of the wall of inferior horn and signal changes in the right mesial-temporal lobe and hippocampus were detected by diffusion weighted images (DWI) and fluid-attenuated inversion recovery (FLAIR) images [45]. The findings of the images suggest to the authors a scenario of right encephalitis associated with lateral ventriculitis, pointing out the high potential of COVID-19 to colonize the CNS. 

#### 3.3.5. Acute Myelitis 

In the pandemic epicenter of Wuhan city, an old man presenting with fever and body aches also showed acute myelitis [46]. Authors reported in the present case report the acute flaccid paralysis of bilateral lower limb, urinary and bowel incontinence and sensory level of T-10 when the patient was admitted to the hospital. He presented with both SARS-CoV-2 ribonucleic acid (RNA) in the nasopharyngeal secretion and patchy pneumonia with negative serology for all other infectious diseases. His response to antibiotics and antiviral treatment, intravenous immunoglobulins (IVIG) and steroids was good, and he was isolated for rehabilitation. Cytokine storm and overactive inflammatory response was pointed out by the authors as the cause of acute myelitis, a fact supported by evidence of high levels of several cytokines and other markers in the blood [46]. Regrettably, due to the Wuhan city epidemic, the lack of coronavirus PCR testing in cerebrospinal fluid and MRI imaging of the spine represent the major limitation of this case report, which would have further supported that the acute myelitis was associated with COVID-19 infection.

#### 3.3.6. Headaches and Dizziness 

These two clinical features are nonspecific and minor symptoms of many diseases. Although the pathophysiology with its detailed mechanism has never been discussed and/or postulated, headaches and dizziness have been described to be associated with presentation of COVID-19 as minor symptoms in different reports, however with little incidence [47,48,49,50]. 

### 3.4. Peripheral Nervous System Symptoms of COVID-19 

#### 3.4.1. Anosmia, Ageusia and Chemosensory Dysfunctions

In the USA, an internet-based cross-sectional survey documented chemosensory dysfunction in COVID-19-positive patients [51,52]. Indeed, COVID-19-positive people presented a loss of smell and/or taste when compared to healthy subjects. Interestingly, in this study, no need for hospitalization and mechanical ventilation was required by the patients who were mostly ambulatory. Authors try to explain this fact by the transmission route of COVID-19, which is likely to be nasal and buccal for ambulatory patients in respect to a preferential pulmonary route for hospitalized people. This is indeed indicative of a possible inhibition of chemosensory detection by local inflammation [30,31]. At the same time, in a large Iranian cohort, a similar situation was found with an online questionnaire-based survey [53,54]. Most participants presented with a decreased sense of smell and some of them also had a decreased taste sensation, reporting flu or cold symptoms before anosmia, headaches, nasal stiffness and fever. On the other hand, the study by Mao et al. reported very few cases with an impairment of taste and smell in patients from the Chinese cohort described above [23] and fewer of these symptoms were reported in the French cohort by Helms and colleagues [24].

#### 3.4.2. Guillain–Barré Syndrome (GBS) 

China, Iran, USA and Italy reported eight cases of GBS associated with COVID-19 so far. An old woman travelling to Wuhan City was the first case [50]. Nerve conduction studies (NCS) and electromyography (EMG) conducted for this woman showed demyelinating polyneuropathy with acute weakness in both legs and severe fatigue before the appearance of respiratory manifestation and viral particles in the nasopharyngeal secretions [55]. Based on the travel history, and clinical situation due to COVID-19, authors identified a para-infectious pattern of GBS. Similarly, an old man with diabetes from Iran developed ascending paralysis after hospitalization with classical respiratory symptoms of COVID two weeks before [56]. 

Acute motor sensory axonal neuropathy was also suggested by NCS/EMG. Although still no direct link has been found, given the respiratory impairment in COVID-19, which can result in immunological damage of the peripheral nervous system, GBS might represent a new neurological complication of COVID-19. Another GBS case was reported in the USA [57]. The patient tested positive for COVID-19, and displayed ascending paralysis leading to respiratory difficulty. 

Five patients with GBS associated with COVID-19 were reported in Northern Italy, presenting features of ataxia, paresthesia, and lower-limb weakness [58]. Patients showed demyelinating and/or axonal polyneuropathy and all tested positive for SARS-CoV-2 detection by a nasopharyngeal swab.

These series of reports clearly indicate an association between COVID-19 and GBS but, as previously mentioned, to finally prove if a causal relationship exists [59,60], further large-scale studies are still required.

#### 3.4.3. Skeletal Muscle Damage and Others

The Chinese cohort described by Mao and colleagues reported a higher percentage of skeletal muscle injury in COVID-19-positive patients [23]. The criteria used to define this condition were myalgia and elevated serum creatine kinase level (above 200 U/L) thought to be a complication due to the cytokine storm. However, it is difficult to exclude the idea that the presence of skeletal muscle damage might be secondary to myopathy and neuropathy since numbers are still too low to provide a clear picture [61,62]. In the same study, authors also reported neuralgia epilepsy and ataxia, unfortunately without providing further details [23].

## 4. Discussion

The epidemic model based only on respiratory droplets and close contact could not fully explain the regional differences in the spreading of the COVID-19 in many countries. There is increasing evidence concerning the association between particulate matter concentrations and COVID-19 mortality, therefore several scientists are proposing the hypothesis that air pollution could play a role in the progression of the COVID-19 epidemic. Several studies have addressed the role played by the airborne transmission route and particulate matter (PM) in the spreading of viral infections [63,64,65]. Ambient influenza A virus was shown to be significantly higher during the Asian dust days than during the background days [13]. Additionally, respiratory syncytial virus (RSV) was demonstrated to cause pneumonia in children by its penetration in the deepest parts of respiratory apparatus, promoted by particle-based transport, and a positive correlation between the infection rate and the particulate matter PM2.5 (r = 0.446, *p* < 0.001), PM10 (r = 0.397, *p* < 0.001) was shown [66]. Additional evidence comes from data about the association between daily numbers of measles cases and PM2.5 concentrations observed in 21 cities in China during the epidemic that occurred from October 2013 to December 2014 [14]. The authors of this latter study highlighted that a detectable increase in PM2.5 by 10 µg/m^3^ was significantly associated with raised measles incidence. Therefore, researchers concluded with the final recommendation to adopt serious PM reduction strategies in order to slow down the measles infection diffusion rate [14]. Finally, Peng et al. in 2020 demonstrated that PM concentration levels in China significantly affected the spreading of measles outbreaks in Lanzhou [67]. Additionally, in this case, the authors suggested developing effective abatement strategies of PM outdoor concentrations in order to reduce potential health risks for the population. After the spreading of COVID-19 at the beginning of 2020, Van Doremalen et al. have proven the higher aerosol and surface stability of SARS-CoV-2 as compared with SARS-CoV-1, with the virus remaining viable and infectious in aerosol for hours [68]. Paules et al. (2020) highlighted that airborne transmission of SARS-CoV can occur, in addition to close distance contact [69]. Finally, research carried out in the USA by Xiao et al. seems to confirm an association between increases in particulate matter concentration and mortality rates due to COVID-19 [22]. Further experimental studies could assess the possibility that particulate matter may act as a “carrier” for the viral droplet nuclei, as it has been shown for other viruses, eventually impressing a boost effect to the spreading of the viral infection.

The primary targets of COVID-19 are the respiratory system (causing symptoms such as cough, dyspnea, sore throat, lung deficiency) and cardiovascular system (impaired heart function and coagulation disorders), which are already impaired by the long term exposures to high levels of particulate matter, possibly resulting in a more severe clinical course of infections acquired by debilitated individuals, especially in most vulnerable age groups. In addition to respiratory and cardiovascular systems, neurological involvement has been shown to be quite common and, if not highlighted and treated in reasonable time, may cause serious complications. The central and peripheral nervous systems are both part of the spectra of neurological alterations caused by COVID-19. In this review, we performed a literature search in order to provide a characterization of neurological symptoms and complications associated with COVID-19. Peripheral nervous system manifestations, particularly anosmia and ageusia, are probably the most common and earliest symptoms of COVID-19 disease, with mild or severe smell and/or taste dysfunctions often persisting even for months after recovery [51,52]. Other peripheral symptoms (i.e., Guillain–Barré Syndrome (GBS), skeletal muscle damage and myopathies) as well as central nervous system manifestations are less frequent but more severe and include headaches, dizziness, epilepsy, encephalopathy, acute hemorrhagic necrotizing encephalopathy (ANE), cerebrovascular accidents (CVA), encephalitis and acute myelitis [36,37,38,39,40,41,42,43,44,45,46,47,48,49,50]. Severely ill patients usually present with the majority of symptoms. However, in some COVID-19 patients, neurological alterations either preceded respiratory symptoms or even were reported as the sole symptoms. Therefore, while dealing with these “neurological” COVID-19 cases, appropriate treatment and prevention can be achieved only by employing an attentive examination of the patient. It is also important that the neurological manifestations from different parts of the world are systematically analyzed and reported in the literature, in order to completely and properly document the evolution of these symptoms or complications. 

Most reports of COVID-19 clinical manifestations including complications are based on clinical studies from tertiary centers. To understand the etiology and the impact on public health, especially considering the role of pollution, we need more population-based data. For these reasons, the present outbreak calls for the introduction of surveillance systems to monitor changes in the frequency of neurological and cardiovascular diseases within COVID-19 infection population data [70,71].

## 5. Conclusions

In addition to respiratory symptoms, central and peripheral nervous system manifestations are frequent in COVID-19 patients. Self-reported anosmia and ageusia are key symptoms of the coronavirus disease and may persist even after the acute infective phase of COVID-19 disease. Other mild or severe clinical features directly affecting the brain or nerves have been reported in medical literature since the beginning of the epidemic. Therefore, neurological symptoms and complications associated with COVID-19 should be considered as part of the clinical features of this novel global pandemic.
